# How Effective Is Saline Solution Infiltration During Coaxial Needle Withdrawal in Reducing the Incidence of Pneumothorax in CT-Guided Lung Biopsies?

**DOI:** 10.3390/diagnostics16193080

**Published:** 2026-09-22

**Authors:** Giulia Viviano, Ganyat Adenike Ralitsa Adebanjo, Giorgio Concari, Chiara Martini, Giuseppe Pedrazzi, Miltos Krokidis, Massimo De Filippo

**Affiliations:** 1 University Hospital of Parma, Via Gramsci 14, 43126 Parma, PR, Italy; 2 Areteion Hospital, National and Kapodistrian University of Athens, 76, Vas. Sophias Ave, 11528 Athens, Greece

**Keywords:** pneumothorax, lung biopsy, saline solution

## Abstract

**Background/Objectives**: This study evaluates whether saline infiltration during coaxial needle withdrawal reduces the incidence of pneumothorax (PTX) in CT-guided lung biopsies, potentially enhancing procedural safety. **Methods**: A retrospective analysis of 200 consecutive CT-guided core needle biopsies (18G) was conducted using our hospital’s Radiology Department database. All procedures were performed between February 2022 and December 2024 by a single operator with 20 years of experience. Patients were divided into two groups: Group A (100 patients), receiving 10 mL of 0.9% saline solution during coaxial needle withdrawal, and Group B (100 patients), in whom no saline was infiltrated. Descriptive and inferential statistical analyses were used to assess the relationship between saline infiltration and the incidence of PTX. **Results**: PTX occurred in 24% of Group B patients versus 7% in Group A (*p* = 0.001). Multivariate analysis identified saline infiltration as a significant protective factor, even in high-risk patients (e.g., those with emphysema or in lateral decubitus). The odds ratio (OR = 0.18; 95% CI: 0.07–0.48) indicates that saline infiltration significantly reduces the risk of PTX. **Conclusions**: Infiltration of 10 mL of saline during coaxial needle withdrawal significantly reduces the incidence of PTX in CT-guided lung biopsies, offering a simple and effective strategy to enhance patient safety.

## 1. Introduction

Lung cancer remains the leading cause of cancer-related mortality worldwide, with more than two million new cases diagnosed each year and a mortality rate that exceeds the combined deaths from breast, colorectal, and prostate cancers. Prognosis remains strongly dependent on the stage at diagnosis: while tumors detected at an early stage may have a 5-year survival rate exceeding 60–70%, survival in advanced disease remains below 10%. For this reason, recent years have seen growing interest in strategies aimed at early detection and biological characterization of pulmonary lesions [1].

The widespread implementation of screening programs using low-dose computed tomography (LDCT) has led to a significant increase in the detection of incidental or suspicious pulmonary nodules, making the need for definitive histological diagnosis increasingly common. In this context, the accurate diagnostic assessment of pulmonary nodules represents a crucial step in the clinical pathway, as it allows the differentiation of benign lesions from early-stage neoplasms, whether primary or metastatic, thereby guiding subsequent therapeutic decisions.

The diagnostic workup of a suspicious pulmonary lesion relies on a combination of clinical, radiological, and histopathological findings. Morphological features on CT—such as lesion size, spiculated margins, the presence of solid or subsolid components, growth pattern, and growth rate—may suggest the nature of the lesion; however, in most cases they are insufficient to establish a definitive diagnosis. Therefore, histological confirmation remains the gold standard for determining the nature of the lesion and guiding oncologic management.

From a pathological perspective, lung cancer is broadly classified into two main categories: small cell lung cancer (SCLC) and non-small cell lung cancer (NSCLC), which differ substantially in terms of biological behavior, prognosis, and therapeutic management. NSCLC accounts for approximately 85% of all lung cancers and includes several histological subtypes, most commonly adenocarcinoma, squamous cell carcinoma, and large cell carcinoma. In contrast, SCLC is characterized by a more aggressive clinical course and is typically treated with systemic chemotherapy and radiotherapy rather than targeted therapeutic approaches [2].

In recent years, the increasing use of targeted therapies and immunotherapy has further highlighted the importance of adequate tissue sampling. Molecular characterization of lung cancer, including the identification of driver mutations such as *EGFR*, *ALK*, *ROS1*, *BRAF*, or *MET*, now represents an essential step in selecting the most appropriate therapeutic strategy. This requires the availability of adequate tissue samples not only for histological diagnosis but also for genomic and molecular analyses [3,4].

Over the past two decades, non-small cell lung cancer has progressively become a paradigm of precision medicine in oncology. The identification of specific molecular alterations has enabled the development of drugs capable of selectively targeting the oncogenic pathways responsible for tumor growth. Activating mutations in the *EGFR* gene and rearrangements involving the *ALK* and *ROS1* genes represent well-established therapeutic targets. In patients harboring these alterations, treatment with tyrosine kinase inhibitors (TKIs) has demonstrated significantly improved clinical outcomes compared with conventional chemotherapy [5].

Concurrently, the introduction of immunotherapy has further expanded the available therapeutic options. Biomarkers such as programmed death-ligand 1 (PD-L1) expression and other characteristics of the tumor microenvironment play an important role in predicting response to immune checkpoint inhibitors, which have shown substantial survival benefits in patients with advanced disease. Current international guidelines therefore recommend routine molecular testing using extended biomarker panels in patients with advanced NSCLC to identify actionable alterations and guide personalized treatment strategies [6].

In this context, CT-guided percutaneous lung biopsy represents one of the most widely used techniques for obtaining tissue samples from peripheral pulmonary lesions, enabling their histological, genotypic, and molecular characterization [1].

Additionally, due to its superior diagnostic accuracy and lower costs, CT-guided lung biopsy is often preferred over bronchoscopy [7,8].

Lung biopsy using a cutting needle is a minimally invasive procedure that allows tissue sampling from a target lesion through the insertion of an 18–21 G needle under CT guidance. This approach ensures precise sample collection while minimizing potential damage to surrounding structures [8].

The use of coaxial systems allows multiple samples to be obtained through a single pleural access, thereby reducing parenchymal trauma and improving the quality of the collected specimen.

Despite its minimally invasive nature, percutaneous lung biopsy is not free from complications. The most common are pneumothorax and parenchymal hemorrhage, with variable incidences reported in the literature.

A review by Chockalingam and Hong, which examined complications following CT-guided transthoracic biopsy with a fine needle (22G), reported a pneumothorax (PTX) incidence of up to 61% and a parenchymal hemorrhage incidence of up to 11% [8,9,10,11].

Numerous factors have been associated with an increased risk of pneumothorax during CT-guided lung biopsies. These include the presence of emphysema, a greater distance between the lesion and the pleura, the number of needle passes through the pleural cavity, the duration of the procedure, and the patient’s position during the examination. Needle gauge and the type of biopsy system used may also influence the incidence of complications.

Consistent with the literature, parenchymal hemorrhage following lung biopsy is generally of minimal clinical significance and is associated with a reduced incidence of pneumothorax on post-procedural CT scans [8].

This phenomenon may be explained by the fact that blood itself fills the air space left by the coaxial needle during its withdrawal and exerts a “sealing” effect between the parietal and visceral pleura, promoting their adhesion and interfering with the accumulation of air in the pleural cavity.

Based on this pathophysiological observation, several techniques have been proposed in recent years to reduce the risk of pneumothorax, including the autologous blood patch, the injection of a gelatin sponge, or the use of saline solutions along the needle tract. These strategies aim to facilitate closure of the pleuroparenchymal tract at the end of the procedure, thereby limiting communication between the alveoli and the pleural space.

Considering this rationale, we sought to reproduce the same effect by injecting 10 mL of saline solution during withdrawal of the coaxial needle at the end of the procedure.

The aim of this study was therefore to evaluate whether infiltration of 10 mL of 0.9% saline solution during coaxial needle withdrawal in CT-guided lung biopsies reduces the incidence of pneumothorax, and to analyze the role of other clinical and procedural factors associated with the development of this complication.

## 2. Materials and Methods

### 2.1. Patients

Patients who underwent CT-guided percutaneous lung biopsy at the University Hospital of Parma between February 2022 and December 2024 were retrospectively identified. They were divided into two groups: Group A (100 patients), in whom 10 mL of 0.9% saline solution was injected during coaxial needle withdrawal, and Group B (100 patients), in whom no saline solution was used.

The database included only patients with focal lung lesions (nodules or masses) who had either undergone non-diagnostic bronchoscopy or for whom bronchoscopy was not indicated. All consecutive patients meeting these criteria during the study period were included, without predefined exclusion criteria, in order to reflect routine clinical practice. No predefined exclusion criteria related to previous ipsilateral procedures, pleurodesis, pleural adhesions, or emphysema were applied. In the study cohort, no patients had a history of pleurodesis or clinically relevant pleural adhesions. Apart from emphysema, which was specifically assessed and did not represent an exclusion criterion, no relevant pre-existing pleural or pulmonary conditions potentially affecting pneumothorax risk were encountered. Anticoagulant therapy was temporarily discontinued before the procedure as part of routine pre-procedural management, whereas antiplatelet therapy was generally maintained. No patients in the analyzed cohort had clinically relevant coagulation abnormalities precluding the safe performance of the biopsy.

Saline infiltration was progressively introduced into routine clinical practice during the study period. Its use became increasingly frequent from 2023 onward and was performed almost routinely thereafter. However, the adoption of the technique was not strictly determined by calendar date: saline infiltration was occasionally performed before 2023, whereas some procedures without saline were also performed after its introduction into routine practice. Therefore, the two study groups were not strictly sequential and did not correspond exclusively to pre-2023 and post-2023 periods.

The sample size was determined by the number of consecutive eligible patients during the study period.

The study received approval from the local Ethics Committee.

### 2.2. Procedure

A single operator with 20 years of experience in interventional radiology (M.D.F.) performed all CT-guided lung biopsy procedures included in this study. The procedures were conducted using a dual-source 128-slice CT scanner (Somatom Definition Flash, Siemens Healthineers, Forchheim, Germany) under sterile conditions, with local anesthesia (subcutaneous injection of 5–6 mL of 1% lidocaine).

The needle pathway through the lung parenchyma was carefully planned at the start of each procedure using CT images and three-dimensional reconstructions. Tissue samples were obtained using an 18-gauge cutting needle (HistoCore, Solna, Sweden) introduced through a 16-gauge coaxial needle.

The patient’s position on the CT table—whether prone, supine, or lateral—was determined by the location of the target lesion. A metal grid served as a landmark to identify the needle entry point. To allow rapid assessment, a cytologist was present on site to evaluate the cellular content of the collected sample, allowing the radiologist to verify successful sampling or adjust the needle for improved targeting of the lesion if needed.

In Group A, 10 mL of saline solution was gradually infiltrated during coaxial needle withdrawal at the end of the biopsy procedure. The saline was injected slowly and continuously, starting as the coaxial needle was withdrawn and continuing until it had been completely removed from the skin.

All patients underwent a CT scan immediately after the procedure, followed by a chest X-ray approximately two hours later to assess for potential complications such as pneumothorax.

### 2.3. Data Collection

CT and X-ray images were retrieved from our Picture Archiving and Communication System (PACS) and independently reviewed by two readers. Cases were considered positive for pneumothorax if PTX was evident on both the post-procedural CT scan and the X-ray taken two hours after the procedure, or if clinically significant PTX required percutaneous drainage. Millimeter-sized pneumothoraces detected on post-procedural CT scans but no longer visible on the two-hour follow-up chest X-ray were not classified as PTX, as they were considered clinically insignificant and without impact on patient management or hospital stay.

Additional variables analyzed included the presence and severity of emphysema, assessed on pre-procedural CT as mild (≤25%), moderate (25–50%), or severe (≥50%); the distance between the lesion and the pleura (representing the portion of lung parenchyma traversed by the needle); the duration of the procedure in minutes; the segmental or lobar location of the lesion; the patient’s position during the procedure; and lesion size. These emphysema categories were defined by the authors on the basis of their clinical experience and institutional case series.

### 2.4. Statistical Analysis

A multivariate analysis using binary logistic regression was performed to simultaneously evaluate the association of emphysema, procedure duration, patient positioning, lesion depth, lesion location, and lesion size with the occurrence of PTX. The statistical analysis was conducted using Jamovi (version 2.5.6; https://www.jamovi.org).

For each variable included in the database, appropriate descriptive statistics were calculated, including measures of central tendency (arithmetic mean, median), measures of dispersion (standard deviation, minimum, and maximum), and the distribution of the data. Standard errors and corresponding 95% confidence intervals were also calculated when relevant.

The normality of continuous variables was assessed using the Shapiro–Wilk test separately within each study group. Significant departures from normality were observed for lesion size (Group A: W = 0.903, *p* < 0.001; Group B: W = 0.926, *p* < 0.001), lesion depth (Group A: W = 0.922, *p* < 0.001; Group B: W = 0.807, *p* < 0.001), and procedure duration (Group A: W = 0.792, *p* < 0.001; Group B: W = 0.919, *p* < 0.001).

Correlations between variables of interest were evaluated using Pearson and Spearman correlation coefficients, along with statistical significance tests. Qualitative data were summarized in tables and presented as absolute frequencies, relative frequencies, and percentages.

Graphical analyses, including histograms, scatterplots, and box plots, were used to visualize data distribution and identify potential outliers. Histograms and box plots were particularly helpful in assessing differences between the two study cohorts.

Given the non-normal distribution of the continuous variables, these data were summarized as median and interquartile range (IQR), and between-group comparisons were performed using the Mann–Whitney U test.

Univariate comparisons between categorical variables were performed using the Chi-square test or Fisher’s exact test.

For both continuous and categorical variables, statistical significance was set at *p* < 0.05.

## 3. Results

The baseline characteristics of the study population are summarized in Table 1. No significant between-group differences were observed for lesion size or procedure duration, whereas lesion depth was significantly greater in Group A than in Group B (*p* < 0.001, Mann–Whitney U test).

The incidence of pneumothorax in Group B was 24% (24 patients), whereas in Group A, it occurred in only 7% of cases (7 patients). This indicates a significant association between the occurrence of PTX and the intraprocedural infiltration of 10 mL of saline solution (*p* = 0.001) (Figure 1, Figure 2, Figure 3 and Figure 4). Furthermore, among the 24 patients in Group B, 13 (54%) developed clinically significant PTX requiring percutaneous drainage, compared with 3 of the 7 patients (43%) in Group A.

Among the other variables considered, lesion depth (OR = 1.02; 95% CI: 1.00–1.05; *p* = 0.030) and patient positioning in lateral decubitus (OR = 4.96; 95% CI: 1.52–16.19; *p* = 0.008) were significantly associated with PTX incidence. PTX occurred more frequently with greater needle depth and when patients were positioned in lateral decubitus. A higher incidence of PTX was also observed in patients with emphysema (OR = 1.36; 95% CI: 0.90–2.04; *p* = 0.140) and during longer procedures (OR = 1.01; 95% CI: 0.98–1.05; *p* = 0.458); however, these associations did not reach statistical significance. No significant association was found between PTX occurrence and lesion size or lesion lobar location (Table 2).

The most notable finding was the independent protective effect of saline solution, which significantly reduced the risk of pneumothorax (OR = 0.18; 95% CI: 0.07–0.48; *p* = 0.001). Conversely, the absence of saline was associated with a more than fivefold increase in pneumothorax risk, independent of other clinical and procedural variables.

## 4. Discussion

In the overall sample of 200 patients, the incidence of pneumothorax (PTX) was significantly lower in the group in which saline solution was infiltrated (Group A, 7%), involving only 7 out of 100 patients. In contrast, in the control group in which no saline solution was infiltrated (Group B), 24 patients (24%) developed PTX. This incidence is consistent with data reported in the literature, where pneumothorax rates range from 20% to 60%, with chest tube placement required in approximately 5% to 15% of cases, even when smaller-gauge needles (21–22 gauge) are used compared with the 18-gauge needles employed in the present study [12,13].

The pathophysiological mechanism underlying the protective effect of saline solution is probably that saline infiltrated along the needle withdrawal tract compresses the surrounding pulmonary parenchyma, thereby reducing communication between injured alveoli and the pleural space. This effect may be particularly relevant in the first minutes after the biopsy, when the risk of pneumothorax is highest.

In addition, as with blood, the presence of fluid may promote adhesion between the pleural layers, contributing to the filling of the pleural cavity and preventing the entry of air.

The most relevant finding concerns the volume of saline solution infiltrated. In many of the studies reviewed, the amount of saline did not exceed 5 mL (Table 3). The higher incidence of pneumothorax reported in these studies is likely attributable to the smaller volume of saline used compared with the 10 mL used in our study. Based on our clinical experience, it is reasonable to hypothesize that volumes of saline below 10 mL are insufficient to promote an adequate filling effect and adhesion between the pleural layers, which is essential to prevent the occurrence of PTX.

As expected, other factors known to be associated with the development of pneumothorax were also observed in our cohort.

Although emphysema did not reach statistical significance in the multivariate analysis, a higher incidence of PTX was observed in emphysematous patients. This may be attributed to the increased air content and reduced parenchymal tissue within the lungs, which can facilitate air leakage and limit the effectiveness of saline infiltration. Among the 7 patients in Group A who experienced PTX, 5 were diagnosed with emphysema: 2 with mild emphysema, 1 with moderate emphysema, and 2 with severe emphysema.

Procedure duration was also associated with a higher incidence of pneumothorax, although this did not reach statistical significance. Longer procedures may reflect greater technical complexity, often related to the location of the lesion or the need for multiple needle repositioning maneuvers. Moreover, prolonged needle dwell time within the pleura may promote the formation of small pleural injuries that facilitate air leakage.

Similarly, patients positioned on their side were more prone to developing PTX. Such cases often involved lesions that were not accessible in other positions (usually prone or supine), making the procedure more challenging and time-consuming because of breathing-induced excursions and the anatomical obstruction posed by the ribs.

Greater lesion depth was also significantly associated with PTX occurrence. A longer intraparenchymal needle tract likely increases the amount of lung parenchyma traversed and, consequently, the probability of alveolar injury and air leakage. At the same time, it may be hypothesized that saline infiltration along a longer tract could exert a more effective sealing effect because a greater volume of parenchyma is available for tract filling; however, this interpretation remains speculative and was not specifically evaluated in our study.

It should be noted that lesion depth was significantly greater in the saline group, whereas greater depth was itself associated with a higher risk of pneumothorax. Therefore, this baseline imbalance would be expected to bias the results against the saline group rather than in its favor. The fact that saline infusion remained independently protective in the multivariate analysis further supports the robustness of this finding.

Despite the presence of these risk factors, multivariate analysis demonstrated that saline solution infiltration maintained an independent protective effect. Saline infusion significantly reduced the risk of pneumothorax (OR = 0.18; 95% CI: 0.07–0.48; *p* = 0.001), corresponding to a more than fivefold increase in risk in the absence of saline. This finding is consistent with the difference observed between the 7 patients in Group A and the 24 patients in Group B, further highlighting its effectiveness.

From a clinical perspective, the routine adoption of this technique could have important implications. The reduction in the incidence of pneumothorax not only improves the safety of the procedure but may also translate into a decrease in hospitalizations, healthcare costs, and patient discomfort.

A further advantage of the proposed technique is its simplicity. Saline infiltration requires only a few seconds, does not entail significant additional costs, and does not require any special devices or materials. It is therefore readily applicable in routine clinical practice.

However, our study has several limitations. First, it is a retrospective single-center study conducted on a relatively limited number of patients. In addition, all procedures were performed by a single highly experienced operator, which may limit the generalizability of the results to other clinical settings.

Another potential limitation is the lack of prospective randomization of patients between the two groups. Prospective randomized studies with larger sample sizes are needed to confirm the effectiveness of this technique.

An additional limitation is the temporal evolution of saline infiltration during the study period. The technique was progressively introduced into routine clinical practice, becoming increasingly frequent from 2023 onward, although its use was not strictly determined by calendar date. Consequently, the saline and non-saline groups do not represent strictly sequential pre-intervention and post-intervention cohorts. Nevertheless, changes in clinical practice over time may have influenced procedural outcomes and cannot be completely excluded as a potential source of confounding. Although all procedures were performed by the same experienced operator, temporal changes in procedural management or other unmeasured factors may have occurred during the study period. This limitation should therefore be considered when interpreting the association between saline infiltration and pneumothorax reduction. Prospective randomized studies would be preferable to minimize this potential source of bias and to confirm the independent effect of saline tract sealing.

Despite these limitations, the results obtained suggest that saline infiltration along the needle tract represents a promising strategy for the prevention of pneumothorax in CT-guided lung biopsies. Moreover, a standard injection volume of 10 mL appears to be effective in most cases.

## 5. Conclusions

In CT-guided lung biopsies performed with an 18-gauge cutting needle, the incidence of pneumothorax is significantly reduced when 10 mL of saline is infiltrated during coaxial needle withdrawal.

Overall, routine saline infiltration during coaxial needle withdrawal should become an integral part of CT-guided lung biopsy and should be performed by all interventional radiologists, as it significantly reduces the incidence of PTX and, consequently, its negative clinical impact on patients, the length of hospitalization, and healthcare costs.

## Figures and Tables

**Figure 1:**
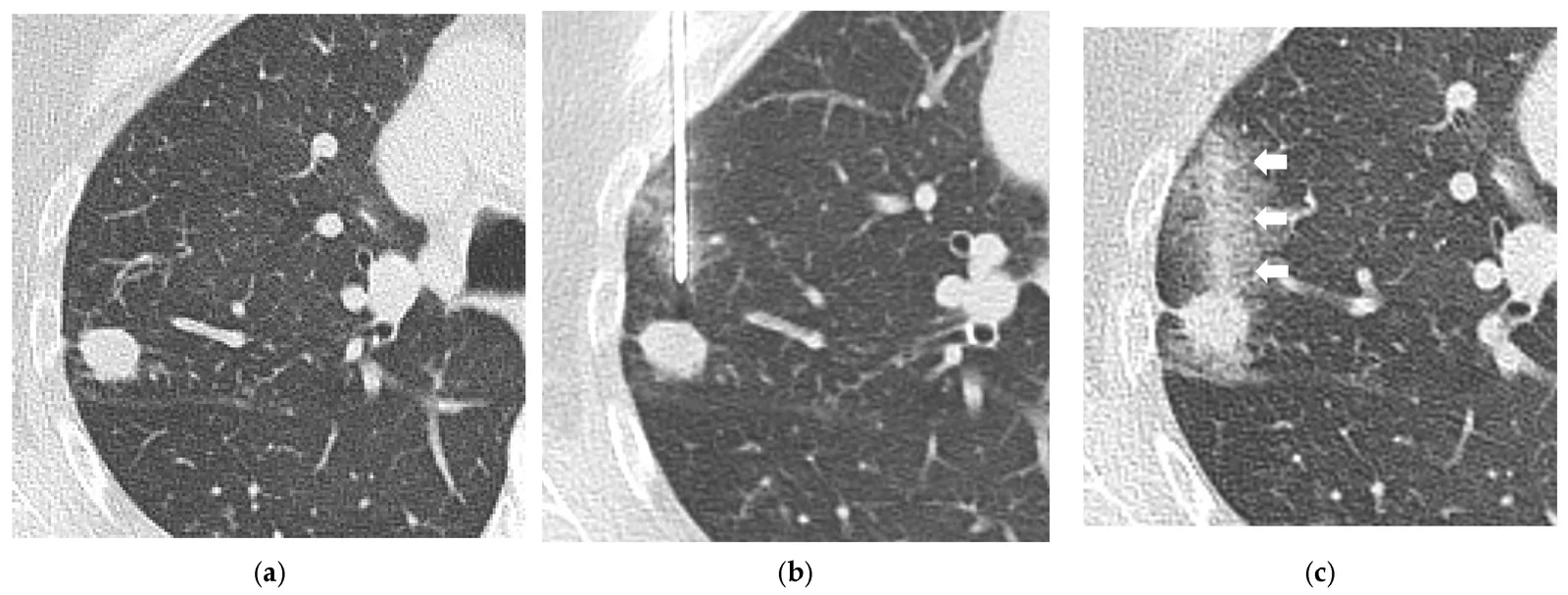
(**a**–**c**). Chest CT images of a 78-year-old woman acquired before (**a**), during (**b**), and after (**c**) the infiltration of 10 mL of saline solution during coaxial needle withdrawal in a CT-guided 18G core needle lung biopsy. Histological examination revealed an inflammatory lesion. (**a**) A 14 mm solid, non-calcified nodule in the middle lobe. (**b**) Coaxial needle positioned within the lung parenchyma. (**c**) Post-procedural CT image showing a blurred, hyperdense tract caused by saline (NaCl) infiltration, without pneumothorax.

**Figure 2:**
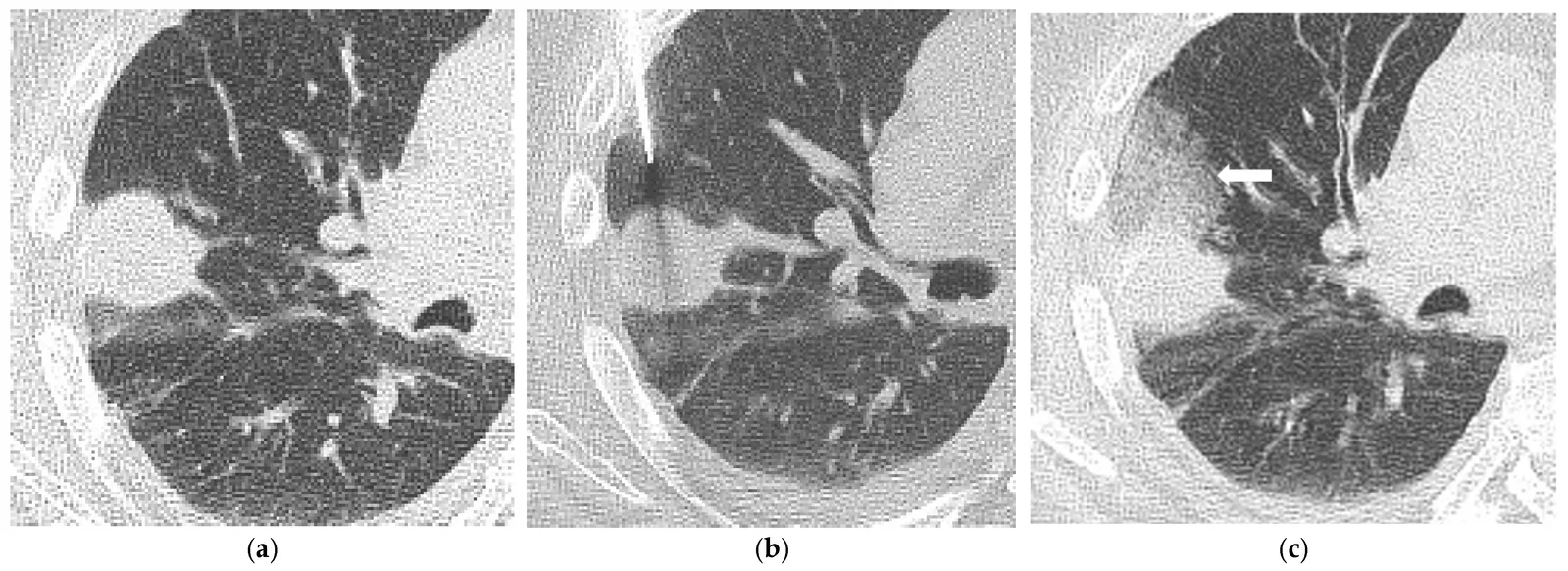
(**a**–**c**). Chest CT images of a 69-year-old man acquired before (**a**), during (**b**), and after (**c**) the infiltration of 10 mL of saline solution during coaxial needle withdrawal in a CT-guided 18G core needle lung biopsy. Histological examination confirmed a metastasis from thyroid carcinoma. (**a**) A 31 mm rounded lesion with spiculated margins in the right upper lobe. (**b**) Coaxial needle positioned within the lung parenchyma. (**c**) Post-procedural CT image showing a blurred, hyperdense halo caused by saline (NaCl) infiltration, without pneumothorax.

**Figure 3:**
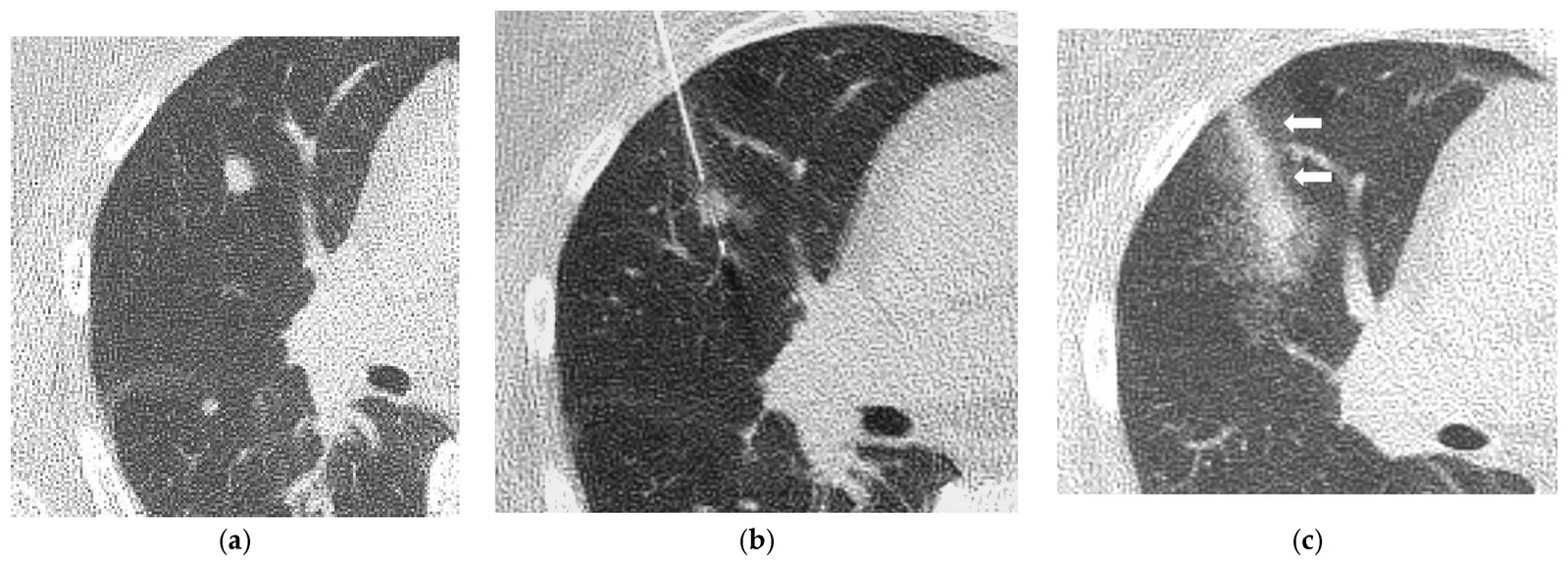
(**a**–**c**). Chest CT images of a 60-year-old man acquired before (**a**), during (**b**), and after (**c**) the infiltration of 10 mL of saline solution during coaxial needle withdrawal in a CT-guided 18G core needle lung biopsy. Histological examination confirmed an inflammatory nodule. (**a**) A 12 mm solid lung nodule in the middle lobe. (**b**) Coaxial needle positioned within the lung parenchyma and also through the lesion. (**c**) Post-procedural CT image showing a blurred, hyperdense path caused by saline (NaCl) infiltration, without pneumothorax.

**Figure 4:**
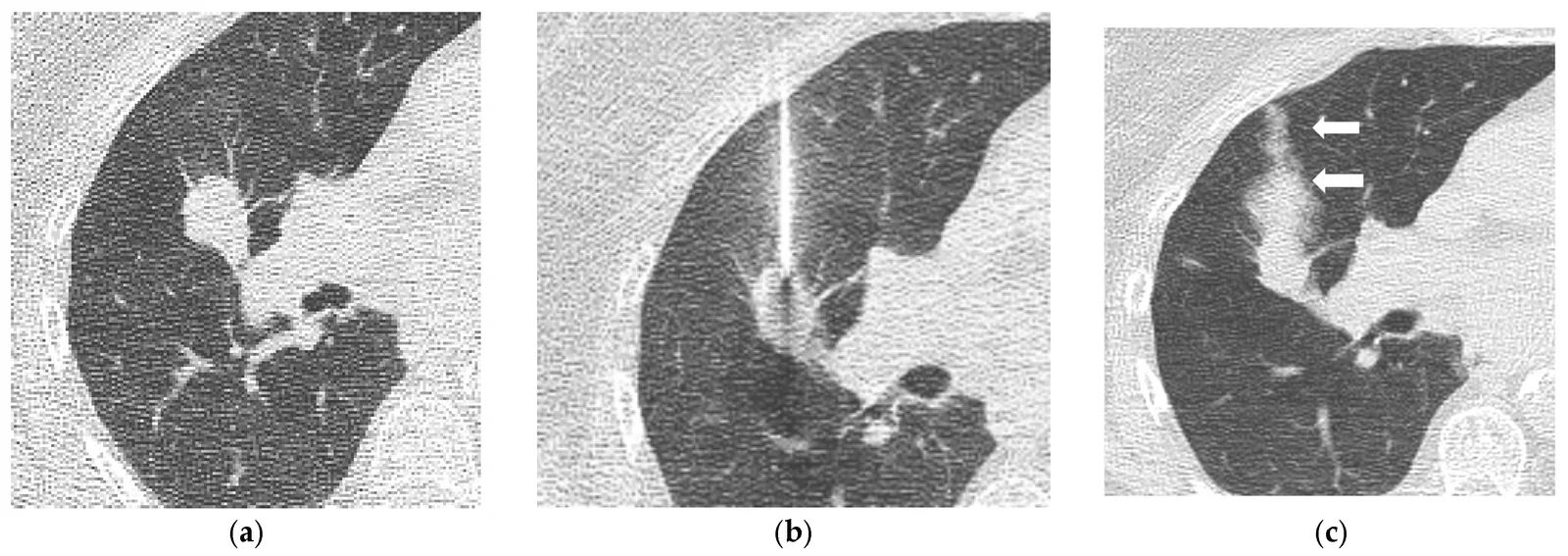
(**a**–**c**). Chest CT images of an 81-year-old woman acquired before (**a**), during (**b**), and after (**c**) the infiltration of 10 mL of saline solution during coaxial needle withdrawal in a CT-guided 18G core needle lung biopsy. Histological examination confirmed a metastasis from rectal carcinoma. (**a**) A 30 mm periscissural nodule with spiculated margins in the middle lobe. (**b**) Coaxial needle positioned within the lung parenchyma. (**c**) Post-procedural CT image showing a blurred, hyperdense path resulting from saline (NaCl) infiltration, without pneumothorax.

**Table 1:** Baseline characteristics of patients undergoing CT-guided lung biopsy.

Variable	Group A (Saline)	Group B (No Saline)	*p*-Value
Lesion size (mm)	33.0 (19.0–45.25)	36.0 (19.75–54.5)	0.278
Lesion depth (mm)	22.0 (11.5–38.0)	9.0 (0–23.0)	<0.001
Procedure duration (min)	20.0 (17.0–25.25)	23.0 (17.0–30.0)	0.190
**Emphysema, n (%)**			0.21
Absent	37 (37%)	48 (48%)	
Mild	39 (39%)	28 (28%)	
Moderate	15 (15%)	14 (14%)	
Severe	9 (9%)	10 (10%)	
**Patient position, n (%)**			0.19
Prone	59 (59%)	55 (55%)	
Supine	36 (36%)	32 (32%)	
Lateral decubitus	5 (5%)	13 (13%)	
**Lesion location, n (%)**			0.58
Upper lobes	50 (50%)	52 (52%)	
Middle lobe/lingula	5 (5%)	6 (6%)	
Lower lobes	45 (45%)	42 (42%)	

Values are expressed as median (interquartile range) for continuous variables and as number (percentage) for categorical variables. Between-group comparisons were performed using the Mann–Whitney U test for continuous variables and the Chi-square test or Fisher’s exact test for categorical variables, as appropriate.

**Table 2:** Multivariate logistic regression analysis of factors associated with PTX.

Variable	OR	95% CI	*p*-Value
Saline use (yes/no)	0.18	0.07–0.48	0.001
Lesion depth (mm)	1.02	1.00–1.05	0.030
Lateral decubitus	4.96	1.52–16.19	0.008
Emphysema	1.36	0.90–2.04	0.140
Procedure duration (min)	1.01	0.98–1.05	0.458
Lesion size (mm)	-	-	>0.05
Lesion lobar location	-	-	>0.05

OR: odds ratio; CI: confidence interval. Variables not reaching statistical significance in the multivariate model are reported with *p*-values only.

**Table 3:** Published articles on 0.9% NaCl infusion to prevent pneumothorax in CT-guided lung biopsies.

Study	Amount of 0.9% NaCl (mL)	N	0.9% NaCl Group vs. Control Group	PTX Incidence (0.9% NaCl Group vs. Control Group)	*p*-Value	Operator(s)
Roman et al., 2024 [14]	3–5	198	87 vs. 111	20.7% vs. 35.1%	0.02	Operator experience is not specified
Satomura et al., 2024 [15]	1–5	303	173 vs. 130	23% vs. 30%	0.177	Single operator with 18 years of experience
Khorochkov et al., 2018 [16]	3–5	278	180 vs. 90	20% vs. 25%	0.05	No info about operator experience
Li et al., 2015 [17]	1–3	322	161 vs. 161	6.2% vs. 26.1%	<0.001	Two experienced thoracic radiologists
Billich et al., 2008 [18]	2–4	140	70 vs. 70	8% vs. 34%	<0.001	Three interventional radiologists (experience of 3–10 years)
Babu et al., 2020 [19]	1–3	200	100 vs. 100	32% vs. 46%	<0.05	One of the five qualified interventional radiologists, >six years of experience
Bourgeais et al., 2024 [20]	3–5	242	93 vs. 149	19.4% vs. 40.9%	<0.001	Both junior and senior radiologists performing the procedure

## Data Availability

The data presented in this study are available on request from the corresponding author due to privacy and ethical restrictions.

## Abbreviations

The following abbreviations are used in this manuscript:

PTX	Pneumothorax
PACS	Picture Archiving and Communication System
